# Supplementary material for: Are carbon emissions trading and green financial instruments synergistic? -Comprehensive quantitative research based on content analysis
Source: PLoS One. 2024 Mar 7;19(3):e0298601. doi: 10.1371/journal.pone.0298601 (PMC10919601; doi:10.1371/journal.pone.0298601)
Supplement: S1 Data — (ZIP) [file pone.0298601.s001.zip › Data/地级市Z_F工作报告.zip!/║╙▒▒/╟╪╗╩╡║╩╨/└┤╫╘╣1⁄2╓┌║┼í╛┬φ┐╦╩2╛▌═°í┐_2017.docx]

各位代表：

现在，我代表市人民政府，向大会报告政府工作，请予审议，并请市政协委员和列席会议的同志提出意见。

一、本届政府工作回顾

2013年以来，在省委、省政府和中共秦皇岛市委的坚强领导下，我们深入贯彻习近平总书记系列重要讲话精神，不断强化政治意识、大局意识、核心意识、看齐意识，落实新发展理念，坚持稳中求进工作总基调，主动适应把握引领经济发展新常态，强力推进供给侧结构性改革，大力实施生态立市、产业强市、开放兴市、文明铸市发展战略，抢抓机遇、攻坚克难、砥砺前行，经济发展总体平稳、向好因素不断积累，社会事业全面进步、人民生活明显改善，推动全面建成小康社会取得新的重要进展，沿海强市、美丽港城建设迈出坚实步伐。

四年来，我们着力稳增长、调结构、转方式，经济建设取得新成绩。生产总值由2012年的1139.4亿元提高到1339.54亿元，人均生产总值由37804元提高到43437元。装备制造、高新技术产业战略支撑作用凸显，电子信息制造业规模超过玻璃制造业，服务业对经济增长的贡献率达到61.1%。城乡发展更趋协调，常住人口城镇化率达到56.13%。全面建成小康社会综合指数居全省前列。

四年来，我们着力激活力、添动力、释潜力，改革开放迈出新步伐。“放管服”、旅游管理体制、城市管理体制等改革取得突破性进展，供给侧结构性改革初见成效。融入京津冀协同发展取得重要成果。抚宁撤县设区，城市发展空间实现跨越拓展。优化投资促进和企业服务工作机制，开放型经济发展成效显著，实际利用外资和进出口总额位居全省前列。全社会创业创新热情日益高涨，北岛博智等一批众创空间释放出蓬勃生机与活力。

四年来，我们着力强基础、补短板、优环境，城市品质迈上新台阶。空气质量优良天数增加72天，PM2.5平均浓度下降29.2%，河流和海域水质持续提升，森林覆盖率达到46.75%，居全省第二。北戴河机场、承秦高速等一批重大交通工程竣工，民族路和北环路上跨铁路桥、兴凯湖路等一批交通节点和断头路打通，城市供水保障、污水处理能力实现质的跃升。全国文明城市、国家卫生城市、国家森林城市“三城同创”使城乡面貌发生可喜变化。

四年来，我们着力办实事、兜底线、惠民生，人民生活实现新改善。市财政累计民生投入708.1亿元，占财政总支出的80%以上，城乡社会保障水平走在全省前列。城镇居民和农村居民人均可支配收入年均分别增长8.6%和9.7%。农村贫困人口减少7.5万，棚户区住房改造3.67万套，农村危房改造3.41万户。建成首批国家公共文化服务体系示范区。荣获全国双拥模范城“六连冠”。各项社会事业统筹推进，人民幸福感逐年提高。

四年来，我们着力转作风、治顽疾、树形象，自身建设呈现新气象。认真开展党的群众路线教育实践活动、“三严三实”专题教育和“两学一做”学习教育，严格落实中央“八项规定”精神，“四风”得到有效整治，工作作风明显转变。坚决落实党风廉政建设“两个责任”，强化对权力运行的监督制约，保持惩治腐败高压态势，风清气正的政治生态进一步形成。

各位代表，2016年是本届政府的收官之年，也是“十三五”开局之年。面对错综复杂的经济形势，面对去产能、治污染、调结构的艰巨任务，我们积极应对、主动作为，统筹推进稳增长、促协同、调结构、惠民生等各项工作，经受住了严峻挑战和考验，克服了诸多困难和风险，经济社会呈现稳中提质、动力增强的良好态势，实现了“十三五”良好开局。

（一）有效应对下行压力，经济运行缓中趋稳、稳中向好。实现生产总值1339.54亿元，比上年增长7%，增速居全省第六位，超过全国、全省增长速度。规模以上工业完成增加值334.87亿元，增长5.2%，增速居全省第六位；规模以上工业企业利润总额34.08亿元，增长71%，增速居全省第二位。固定资产投资完成874.66亿元，保持正增长，环保、新能源投资分别增长1.3倍和47.2%。社会消费品零售总额增长10.4%。一般公共预算收入增长8.9%（可比口径）。城镇居民和农村居民人均可支配收入均增长7.8%。

（二）狠抓供给侧结构性改革，产业转型升级加速推进。全面落实“三去一降一补”重点任务，压减炼铁产能277万吨、炼钢产能75万吨、水泥产能35万吨，超额完成省定任务；商品房去化周期由年初的23个月缩短为14.5个月，进入合理区间；完成215亿元政府性债务置换，在多层次资本市场上市挂牌企业达到50家。开展制造业升级专项行动，规模以上装备制造业、高新技术产业增加值分别增长9.5%和15.1%，总量分别占规模以上工业的45.3%和38.3%。加快发展现代服务业，旅游总收入增长36.7%。成功举办全国康养论坛、煤炭交易大会。推进大众创业、万众创新，建立中小企业贷款风险补偿金，科技型中小企业、高新技术企业分别达到1540家、101家，我市被评为省级小微企业创业创新基地示范城市。农业生产稳中调优，玉米种植压减21.8万亩，创建省级现代农业园区7个，农业产业化经营率69.5%，全市域获批创建省级农业可持续发展试验示范区。

（三）积极主动对接京津，融入协同发展实现突破。北戴河生命健康产业创新示范区获国务院批准成立，成为全国首个国家级生命健康产业创新示范区，获得“一事一议”方式的试点政策支持。示范区总体规划和政策支撑体系编制完成。与北大未名、深圳尚荣、华大基因等一批知名企业、机构签订合作协议36个，北大未名国际健康中心等项目建成运营，与联合国项目事务署共同打造的亚太生命健康产业创新示范区项目全面启动。积极承接京津产业转移和非首都功能疏解，与北控、北汽、新奥、首创等企业集团合作进展顺利，北京医院医联体北戴河分院挂牌，北医三院北戴河国际医院成功落户，国家康复辅具研究中心科研和质检分支机构落成运营，清华大学智能装备研究院等11个项目竣工。京秦高速公路加快推进，京秦高速北戴河新区支线纳入交通运输部“十三五”规划，承秦铁路列入国家中长期铁路网规划，山海关站动车存车场具备存车和整备条件。

（四）持续重拳治理污染，生态环境显著改善。淘汰燃煤锅炉1095台、1314蒸吨，完成工业锅炉改造416台、1687蒸吨，淘汰老旧车辆4740辆，新购置新能源公交汽车366台，严控扬尘和餐饮油烟，克服极端不利的气象条件，PM2.5平均浓度下降4.2%，空气质量优良天数达到281天，全省考核获优秀等次。全面实施“河长制”，开展企业达标排放专项整治、涉水重污染企业退出和水污染防治百日会战，8条主要入海河流水质功能区达标率72.9%，提高13.8个百分点，洋河、戴河、石河恢复III类水质，海水浴场水质全年基本达到国家I类水质标准。完成化学需氧量、氨氮、二氧化硫和氮氧化物年度削减任务。关停露天开采矿山83个，生态修复露天矿山27个。推进国家森林城市创建，完成造林绿化32.85万亩，120万亩储备林建设项目列入国家试点，争取国家、省中长期贴息贷款额度85亿元。青龙列入国家重点生态功能区。

（五）统筹城乡发展建设，综合承载能力不断提升。城市总规修编获国家批准，“多规合一”全面启动。强化滨海地区规划管控，实现沿海地带城市设计全覆盖。祖山连接线基本完工，长城旅游公路主路全线贯通，山海旅游大通道基本成形。秦皇岛火车站交通枢纽、宁海大道、文昌路铁路平交道口、先锋路汤河桥等一批困扰多年的工程投入使用。引青济秦三期如期竣工，日供水能力达到50万吨，北戴河西部水厂基本建成，全市供水保障能力大幅跃升。海港区北部、西部污水处理厂试运行，全市污水处理厂基本达到一级A排放标准，日处理能力达到59万吨。改造街巷路30条、老旧小区30个，创建示范物业小区50个，新建、改造街头游园16个。开展“全民洗城”、市容环境综合整治百日攻坚和取缔“三车”专项行动，城市管理水平显著提升。清理拆除违法用地和违法建设10672处、288.51万平方米，拆出了整洁优美的城乡环境，赢得了人民群众的普遍赞誉。创建省级美丽乡村186个，农村垃圾处理长效机制实现全覆盖。石门镇入选首批中国特色小城镇，卢龙县被列为国家新型城镇化综合试点地区。

（六）深化改革扩大开放，发展活力有效释放。“营改增”、水资源费改税等财政体制改革任务全面落实。深化“放管服”改革，向县区下放市本级行政权力228项。整合海洋管理资源，成立市海洋和渔业局。率先推行城市管理综合执法、旅游管理体制改革，国有景区实现所有权、管理权和经营权分离。耀华集团完成企业重组和职工身份置换，企业改革迈出坚实步伐。完成供销社综合改革试点任务。农村土地承包经营权确权登记颁证面积达到98.5%，土地流转率达到29.6%。落实“五证合一、一照一码”登记制度，市场主体总量达到18.3万户。成立公共资源交易中心。完成省级开发区优化整合，行政审批、管理运营机制、人事薪酬制度改革和投融资平台建设全部落实到位，北戴河新区人事制度改革模式在全省推广。设立5000万元招商引资奖励资金，组建投资促进和企业服务中心，成立32个办理组对重点项目全程领办代办，与华侨城、中建材、赛伯乐、中电科等战略投资者签订协议61项。实际利用外资9.04亿美元，增长5%；完成外贸出口额194.91亿元，增长5.6%。

（七）倾心尽力改善民生，人民幸福感获得感进一步增强。完成民生投入209.8亿元，年初确定的为民实事较好落实。全面开展脱贫攻坚，在市财政承贷1亿元的基础上，连续三年每年至少安排5000万专项资金支持青龙脱贫；派驻扶贫工作组109个，实施脱贫工程项目678个、总投资6.9亿元，3.01万人脱贫出列。全年城镇新增就业5.66万人，登记失业率2.81%，转移农村劳动力2.4万人。企业退休职工基本养老金人均增加161.27元/月，城乡居民基础养老金标准提高到80元/月。在全省率先实现城乡居民基本医疗保险市级统筹，形成了以基本医保为基础、大病保险为补充、医疗救助为兜底的医保制度体系。新建中小学6所、幼儿园39所，改扩建164所，193所山区中小学、1.1万余名师生吃上热餐，308所中小学旱厕改造全面实施；河北对外经贸职业学院实现转型，秦皇岛职业技术学院通过国家骨干高职院校验收。实施棚户区改造15523套，完成农村危房改造8841户，3000套公租房全部分配入住。新开通公交线路22条、新增覆盖小区30个。推进公共文化服务体系建设，市博物馆开工建设，中国长城文化研究中心落户我市。成功举办第三届秦皇岛国际马拉松等大型赛事。强化食品药品安全专项整治，狠抓安全生产监管，加强和创新社会治理，社会保持和谐稳定。成功战胜“7·20”特大暴雨洪灾。高标准完成旅游旺季服务保障任务。

（八）推进政府管理创新，行政效率、服务水平明显提高。科学民主决策机制不断完善，地方立法工作卓有成效，政府法律顾问制度全面推行，依法行政能力进一步增强。践行“五到四从四多”工作法，落实干部工作日志等制度，推行“两式两化”等落实机制，完善市县乡村四级政务服务体系，行政效能不断提升。全面完成公车改革，市级“三公”经费下降27.2%。自觉接受人大法律监督、政协民主监督和社会监督，办理国家、省和市人大代表建议、政协提案536件，按时办复率和规范化率均达100%。全面推进政府系统从严治党，认真履行主体责任，深入整治“四风”，加大违法违纪案件查处力度，一批腐败分子得到应有惩处。

同时，我们扎实开展国防动员和双拥共建工作，积极推进军民融合发展，驻秦部队和广大民兵在森林防火、抗洪抢险等急难险重任务中作出了突出贡献。民族宗教、新闻广电出版、外事侨务、人民防空、方志档案、气象地震、审计、统计、援疆、妇女儿童、老龄、残疾人等事业都有了新的发展和进步。

各位代表！四年走过的道路不同寻常，取得的成绩殊为不易。这是市委总揽全局、正确领导的结果，是市人大、市政协及社会各界大力支持的结果，是市政府历任组成人员团结奋斗，各县区、各部门和全市人民共同努力的结果。在此，我代表市政府向全市人民，向人大代表、政协委员，向各民主党派、工商联、无党派人士和人民团体，向驻秦人民解放军、武警官兵和公安政法干警，向中省直机关驻秦单位，向关心支持秦皇岛发展的海内外朋友们，致以崇高的敬意和衷心的感谢！

各位代表，我们必须清醒地看到，我市经济社会发展仍面临许多矛盾和问题。经济下行压力依然较大，经济总量小、发展速度慢、发展后劲不足的局面尚未根本改观，部分经济指标未达到预期，与省内及沿海先进地区发展的差距仍然较大；产业结构性矛盾仍旧突出，去产能任务繁重，创新能力不强，新技术、新业态、新模式发展相对滞后；环境资源形势依然严峻，个别河流、个别河段污染较重，重污染天气时有发生，环境保护和生态治理任务艰巨；城镇化质量水平尚待提高，城乡和区域发展还不平衡，城市功能不完善，精细化管理水平不高，背街小巷整治、老旧小区物业管理、“双违”打击等任务仍然繁重；社会建设存在薄弱环节，劳动就业、社会保障、收入分配、教育卫生、公共服务等方面与群众期盼仍有较大差距，脱贫攻坚还面临不少难啃的“硬骨头”；政府自身建设仍需加强，一些部门和工作人员适应把握引领经济发展新常态的能力缺乏，慢作为、不作为、乱作为现象仍有发生，懒政怠政、推诿扯皮、消极腐败问题依然存在。对此，我们必须高度重视，切实采取有效措施，认真加以解决。

二、今后五年目标任务

各位代表，刚刚闭幕的中国共产党秦皇岛市第十二次代表大会，精准指明了秦皇岛未来发展的目标任务，科学描绘了宏伟蓝图，开启了建设沿海强市、美丽港城和国际化城市的新征程。面对历史重任和光荣使命，我们必须进一步强化机遇意识、拼抢意识，坚定信心、咬紧牙关、埋头苦干，在新的历史征程中赢得主动、赢得优势、赢得未来。

今后五年经济社会发展的总体要求是：全面贯彻党的十八大和十八届三中、四中、五中、六中全会精神，以邓小平理论、“三个代表”重要思想、科学发展观为指导，深入学习贯彻习近平总书记系列重要讲话精神，统筹推进“五位一体”总体布局，协调推进“四个全面”战略布局，坚持稳中求进工作总基调，牢固树立和落实新发展理念，适应把握引领经济发展新常态，坚持以提高发展质量和效益为中心，以推进供给侧结构性改革为主线，深入实施生态立市、产业强市、开放兴市、文明铸市发展战略，继续解放思想、艰苦奋斗，坚定走加快转型、绿色发展、跨越提升新路，加强预期引导，深化创新驱动，全面做好稳增长、促改革、调结构、惠民生、防风险各项工作，保持经济平稳健康发展和社会和谐稳定，为全面建成小康社会，建设沿海强市、美丽港城和国际化城市而团结奋斗。

按照市第十二次党代会描绘的宏伟蓝图，全面建成小康社会，建设沿海强市、美丽港城，朝着国际化城市迈进，是我市即期、中期、远期发展的三个重大目标台阶。围绕宏伟蓝图的实现，今后五年主要奋斗目标是：“一个全面建成、四个走在前列、五个明显提升”，就是确保2020年如期全面建成小康社会，经济增长速度、质量效益提升幅度、生态改善程度明显高于京津冀城市群平均水平，“十三五”时期经济增速保持在8%以上，争取到2019年生产总值比2010年翻一番，城乡居民人均可支配收入比2010年翻一番，青龙满族自治县如期脱贫摘帽；确保生态环境、城乡统筹、开放水平、城市文明走在京津冀城市群前列；确保发展质量、改革动力、人民生活、社会治理能力、自身建设水平明显提升。

建设沿海强市、美丽港城，力争在2030年左右实现。沿海强市，体现在经济实力强，人均地区生产总值、人均公共预算收入、城乡居民人均可支配收入在京津冀城市群中位居前列，成为环渤海现代服务业和临港产业强市；创新动力强，“四大两特”产业快速成长，科技型中小企业和高新技术企业成为经济发展的主要支撑；发展活力强，成为京津冀区域融合创新先行区、营商环境最优城市；城市辐射带动力强，港产城一体化发展格局形成，东北亚物流枢纽的地位得到确立。美丽港城，体现在生态环境美，建成全国生态文明示范区、京津冀城市群生态标兵城市；城乡容貌美，城乡面貌大幅提升，城镇化率高于全国平均水平，城乡基础设施和承载能力全省领先；社会人文美，城乡公共服务水平和均等化大幅提高，社会保障实现全覆盖，人民幸福感安全感显著增强，特色鲜明、奋进向上的城市精神凝聚形成，步入全国文明城市先进行列。

国际化城市，力争在2040年初步实现。体现在开放包容、国际思维成为城市的文化特质，城市设施、功能和服务全面国际化，经济、社会、文化深度融入全球化，休闲旅游、文化交流、医疗康养、贸易口岸等方面在国际市场占有一席之地。

各位代表，实现上述发展目标，依赖于全市上下的万众一心，依靠政府全体人员的奋力拼搏。做好未来五年的工作，应着力把握好以下四点：一是坚持发展第一要务，聚精会神搞建设，心无旁骛抓发展，以推进供给侧结构性改革为主线，引进新上一批符合国家产业导向和环保要求，具有较强带动性和较高综合效益的大项目好项目，挖掘增长潜力，培育发展动力，拓展发展空间，推动经济总量、经济质量效益跨上新台阶。二是坚持以新发展理念为引领，坚决打破惯性思维和传统路径，全面深化改革，加快转变发展方式，真正做到崇尚创新、注重协调、追求绿色、厚植开放、深谋共享。三是坚持稳中求进工作总基调，科学把握“稳”与“进”的关系，保持战略定力，坚持问题导向、底线思维，发扬钉钉子精神，以稳强基、以进向好，牢牢掌握工作主动权。四是坚持以人民为中心的发展思想，把保障和改善民生作为经济发展的出发点和落脚点，持之以恒为群众办实事、解难事，把发展硬道理更多体现在增进人民福祉上。

各位代表，今后五年我们要结合市情实际，坚定不移地深入实施生态立市、产业强市、开放兴市、文明铸市战略，强优势、补短板、显特色，走好加快转型、绿色发展、跨越提升新路，为实现秦皇岛赶超崛起、朝着国际化城市迈进奠定坚实基础。

（一）深入实施生态立市战略，为走好发展新路筑牢绿色支撑。始终把生态作为发展之本，以打造生态文明先行示范区为目标，将生态文明理念融入经济社会发展各方面，划定生态红线，严守开发边界，实行生态环境损害责任终身追究制度，完善市县乡村四级环保网格化监管体系，以刚性要求促进生态保护。以大气、水、土壤污染防治为重点，巩固深化“河长制”，大力实施“碧水行动”“蓝天行动”“净土行动”和山水林田湖海生态涵养修复工程，确保到2020年，主要污染物排放量明显下降，空气质量优良天数比率达到85%，河流水质基本达到功能区标准，北戴河近岸海域水质稳定在I类标准，森林覆盖率达到60%，露天矿山采掘业全部退出，完成国家森林城市、国家卫生城市创建任务。推进生态与产业深度融合，培育绿色低碳循环产业，加快生产生活方式和消费模式绿色转型，打开“绿水青山就是金山银山”的转化通道，让生态美起来、产业强起来、百姓富起来。

（二）深入实施产业强市战略，为走好发展新路注入动力源泉。始终把产业作为发展之基，大力推进供给侧结构性改革，坚决完成“三去一降一补”各项任务，做好“僵尸企业”出清和职工分流安置工作；坚持住房的居住属性，引导房地产业持续健康发展；坚持“调旧”和“育新”协同发力，把旅游作为首位产业，把大健康作为核心产业，整合发展大物流产业，积极培育“大智移云”产业，重点发展特色高端制造业，做大做优特色现代农业，加快形成传统和新兴产业相互融合、快速成长的“四大两特”产业新格局，力争到2020年，服务业占比达到55%以上，产业优化度和发展的质量效益提升幅度处于全省前列；以最大的气力和诚意助推实体经济发展，做大做强龙头企业，做精做活中小企业，做多做优“双创”平台和“种子”，培育壮大“铺天盖地”的科技型中小企业和“顶天立地”的高新技术领军企业。深入实施“中国制造2025”行动纲领和“互联网+”行动计划，推动“两化”融合和网络强市、质量强市、制造业强市建设；着力推进科技进步和创新，促进科技与“双创”深度融合，率先进入国家创新型城市行列。

（三）深入实施开放兴市战略，为走好发展新路广聚资源要素。始终把开放作为发展之要，以开放促改革、促创新。以披荆斩棘的勇气和智慧，加快推进有利于引领经济新常态的体制机制和发展方式改革，充分发挥市场配置资源的决定性作用，切实增强对优质生产要素的吸引力、聚集力。以宽广包容的胸怀扩大开放，精准融入京津冀协同发展，推动优质资源与北京教育、医疗、文化、科技等高端要素融合对接、定向疏解；抢抓国家“一带一路”、中蒙俄经济走廊建设机遇，推进招商引资和项目建设实现新突破。以世界眼光规划建设城市，完善基础设施和公共服务，提高城市承载能力；支持县城产城教融合发展，推动各县建设一批特色强镇，促进县域经济增比进位，实现美丽乡村全覆盖；完成城市管理综合执法改革，像“绣花”一样精细化管理城市，力促城市容貌和环境卫生2018年达到全国先进水平；到2020年常住人口城镇化率达到65%以上，城乡一体化发展格局基本形成。

（四）深入实施文明铸市战略，为走好发展新路提供坚强保障。始终把文明作为发展之魂，创建全国文明城市，实现城市品质和财富的整体增值。深入开展中国特色社会主义、中国梦和社会主义核心价值观宣传教育，推动守信联合激励和失信联合惩戒制度化建设，不断凝练、传播具有秦皇岛特色的城市文化和城市气质；巩固全国公共文化服务体系示范区创建成果，完善覆盖城乡、便捷高效的现代公共文化服务体系；健全投入机制，办好民生实事，兜住民生底线，以实实在在的发展变化增进群众获得感；深入推进法治秦皇岛和平安秦皇岛建设，确保城乡网格化管理、精细化服务取得突破，努力让人民过上更有保障、更有尊严、更加幸福的生活。

各位代表，改革发展无坦途，攻坚克难铸辉煌。我们坚信，只要全市上下齐心协力、担当实干、奋勇拼搏，就一定能够战胜前进道路上的各种艰难险阻，一定能够创造出无愧于时代的业绩，一定能够开拓出秦皇岛无限美好的未来！

三、2017年主要工作

2017年是迎接党的十九大胜利召开的重要一年，是落实省第九次党代会、市第十二次党代会精神的第一年，是推进供给侧结构性改革、化解过剩产能最为较劲的一年。做好今年工作，任务艰巨、意义重大。

今年经济社会发展主要预期目标是：地区生产总值增长8%左右；一般公共预算收入增长7%；固定资产投资增长10%以上；社会消费品零售总额增长10%以上，实际利用外资增长5%；万元生产总值能耗下降3.89%；PM2.5年均浓度下降4.3%，地表水水质优良比例达到60%，化学需氧量、二氧化硫、氨氮、氮氧化物减排完成省下达的目标任务；城镇居民和农村居民人均可支配收入分别增长7.5%和8%；居民消费价格指数涨幅控制在3%以内；城镇登记失业率控制在4%以内；森林覆盖率提高3个百分点；常住人口城镇化率提高2个百分点。重点抓好八方面工作：

（一）深化供给侧结构性改革，促进发展动能换挡提速。立足坚决去、主动调、加快转，强化政策措施，争取更大成效。

持续推进“三去一降一补”。落实“产能真去真退”要求，力争全年任务半年完成，压减炼铁产能433万吨、炼钢产能368万吨。按照市场化、法治化方式，稳妥处置僵尸企业，落实好职工安置各项措施。支持城镇居民改善性住房需求，开展好重点地区营销活动，把控好新增房地产土地供应，保持房地产正常的速度和价格。强化政府债务限额管理，争取更多存量债务置换规模；创新融资方式，支持企业到主板上市，年内上市挂牌企业达到70家。严厉打击非法集资和逃废债行为。完善企业直通车服务机制，降低各类交易成本特别是制度性交易成本，使企业轻装上阵。

改造提升传统动能。实施增强制造业核心竞争力三年行动计划，以智能制造为主攻方向，推动“两化”深度融合，推进智能工厂、数字化车间建设。开展工业技改提升专项行动，实施百项重点工业企业技改项目计划，支持中信戴卡、兴龙轮毂、骊骅淀粉等龙头企业实施技术改造和产品升级，助推中铁山桥与省交投深入合作，以新技术、新模式促进传统产业焕发蓬勃生机。

培育壮大新兴优势产业。深化与北汽集团、威卡威等企业合作，加快哈电集团H级燃气轮机、凯盛科技薄膜太阳能电池组件、思泰意达新厂区等项目建设进度，壮大先进装备制造、电子信息等优势产业，年内高新技术产业增加值增长15%左右。加快华夏、茅台等酒庄建设，推动葡萄酒产业向酒庄化、高端化、精品化转变。实施服务业重点行业发展三年行动计划，加快发展工业设计等新业态。支持数字经济加速成长，加快网络提速降费步伐。推进临港物流园区等建设，促进现代物流业大发展、快发展。加速培育海洋生物、海洋高端装备制造等产业和孵化聚集园区，着力开辟海洋经济发展新空间。

发展全域全季全业态旅游。以承办第二届省旅发大会为载体，推动旅游“下海、上山、入村”，打造山海康养旅游度假区，创建国家全域旅游示范区。加快国际康养旅游中心、海洋欢乐谷、老君顶乡村旅游、森林康养小镇、昌黎葡萄小镇、山海旅游铁路等重点项目实施，培育发展海上娱乐、康体养生、红酒休闲等新业态，不断优化产品结构和业态布局，年内完成投资200亿元。做活做优山海关古城，年内达到国家5A级景区验收标准。完成祖山连接线、长城旅游公路等重点道路工程，实现主要交通枢纽、城市和景区的“无缝对接”。加强旅游集散中心、标识引导系统和智慧旅游“一网三平台”建设，强化旅游市场秩序整治和安全监管，切实改善游客体验。多媒体广范围开展宣传营销，打响京东山海康养旅游品牌。办好第二届省旅发大会，是省委省政府交给我们的重要任务，更是我市旅游发展的重大契机。我们要集各方之智，举全市之力，确保举办一届精彩、卓越、震撼的盛会，展示好我市旅游业态的“国际范”，彰显出干部群众的“精气神”。

推进农业供给侧结构性改革。以创建国家农业可持续发展试验示范区为动力，大力发展绿色农业、健康农业，促进农业增效、农民增收、农村增绿。落实藏粮于地、藏粮于技战略，加强农田水利基础设施建设，完成昌黎、卢龙高标准农田建设任务，恢复和改善农田灌溉面积33万亩。优化农业结构，压减籽粒玉米种植20万亩以上，提高特色果品种植和畜禽养殖规模化水平，控制扇贝养殖密度，推广设施蔬菜、食用菌、中药材等高效农业和种养结合等绿色增产模式。坚持质量兴农，狠抓农产品标准化生产、品牌创建和质量安全监管，搞好品牌农产品产销对接和产地交易市场培育。加强农业现代园区建设，年内省级园区达到10家。加快发展农产品加工业，培育冷链物流、连锁配送等新型业态，新建省级休闲农业与乡村旅游示范点3个以上，推进农业与休闲旅游、现代物流、互联网等深度融合，促进农村一二三产业融合发展。深化供销社、农村集体产权、农村土地制度和国有林场综合性改革，完成农村土地承包经营权确权登记颁证任务，争取土地流转率达到32%。新发展农民合作社、家庭农场各100家，创建合作社市级示范社、家庭农场示范场各20家。

（二）持续扩大有效需求，确保经济发展稳中有进。千方百计用好有利因素，推动供给结构与需求结构有效匹配、消费升级和有效投资良性互动，增强对经济增长的持久拉动力。

把项目建设作为主抓手。开展重点项目建设转型升级年活动，深化项目建设定期调度、现场观摩、考核奖惩等长效机制，抓好总投资14亿元的威卡威汽车零部件北方基地、总投资33.7亿元的京能热电等12个省重点建设项目，推进总投资20亿元的泰盛光伏等100个以上市重点建设项目，力争全年重点项目投资额达到固定资产投资的四分之一以上。健全重大项目全程领办代办制，强化跟踪盯办，确保总投资10亿元的华恒生物三期等9个省重点前期项目，总投资68亿元的抚宁抽水蓄能电站等100个市重点前期项目尽快具备开工条件。强化项目储备动态管理，深入研究宏观政策、产业政策和投资导向，重点谋划一批增后劲、管长远、带全局的大项目好项目，形成项目建设梯次推进格局。推广政府和社会资本合作（PPP）模式，全市所有污水处理厂实现社会化管理和运营。

推动消费加快升级。进一步加大对城市公共设施和旅游景区基础设施建设的支持力度，重点支持县城、棚户区改造、信息基础设施等建设，努力扩大消费新供给。大力实施汽车、居民住房、康养家政服务等“十大扩消费行动”，加强中小商贸流通服务平台建设，拓展信息、绿色、农村等新消费领域。实施“互联网+流通”行动计划，推动实体店销售与网购融合发展，促进电商、快递进社区、进农村。落实职工带薪休假制度，大力发展假日经济。整顿和规范市场秩序，严肃查处假冒伪劣、虚假广告、价格欺诈等行为，让群众消费少烦心、多舒心。

促进外经外贸持续向好。落实鼓励出口的各项政策措施，开展重点外贸企业“一对一”帮扶行动，继续实行出口1美元补贴1分钱政策，鼓励企业扩大出口，确保完成外贸出口额高于上年水平。推进昌黎水产品出口质量安全示范区和省级开发区装备制造出口示范基地建设。支持康泰医学等企业开展跨境电子商务，支持秦冶重工、中铁山桥等企业在境外承揽重大工程，推进秦皇岛加拿大泰瑞斯工业园等境外园区建设，提升“走出去”水平。

大力振兴实体经济。完善政策引导、要素配置和环境保障等有效机制，促进各类资源向实体经济聚集。继续开展企业帮扶活动，深化重点工业企业领导分包责任制，推动“个转企、小升规、规改股、股上市”，新增规模以上工业企业40家，规模以上工业增加值增长6%。深入实施质量强市战略，大力开展“增品种、提品质、创品牌”专项行动，弘扬企业家精神和工匠精神，创建全国质量强市示范市。进一步疏通金融服务实体经济“管道”，防止“脱实向虚”，努力使秦皇岛经济“实”起来、“壮”起来。

（三）深度融入协同发展，努力构建特色发展新优势。坚持服从服务大局、主动融入大局，在对接、服务中加快发展自己。

力促国家级生命健康产业创新示范区开好局起好步。高质量编制核心区规划、产业配套规划等，完善规划体系，优化空间布局；争取国家、省先行先试政策，探索建立示范区法规体系、标准体系、管理体系、金融体系，推进投融资和管理制度创新，吸引商业保险资金参与健康产业发展；深化与华夏幸福、荣盛发展合作，在片区开发、市政工程和生态建设等领域创新建设模式，营造聚集生命健康产业发展的“生态系统”；加强基础设施配套，推进滨海新大道D3段、前程大街等“五纵六横”路网建设；加大项目建设力度，确保冠昊生物等20个项目开工建设，国际生殖中心、吴祖泽院士工作站等项目投入运营，确保年内签约项目50个以上，其中10亿元以上项目超过10个，切实将这一国家级战略平台建设好，为我市跨越赶超提供新动能。

积极打造非首都功能疏解和产业转移承接区。秦皇岛开发区以高新技术产业和先进制造业为主攻方向，以秦皇岛（中科院）技术创新成果转化基地、中关村海淀园秦皇岛分园、北岛博智等承接平台为龙头，加快节能环保产业园等建设，推动与中建材、中电科、中国航天合作项目落地开工，努力在全市产业转型升级和承接京津产业转移中更好发挥示范带动作用。开展开发区改革落实年活动，所有省级以上开发区都设立自己的投融资平台，全面加强基础设施建设、考核机制完善、薪酬制度改革等工作，探索建立支持省级以上开发区承接京津功能疏解和产业转移的政策体系，打造京津冀发展环境最优园区。

推进全国综合性交通枢纽城市建设。启动实施秦皇岛火车站北广场建设工程，深化与北京铁路局、沈阳铁路局战略合作，共建物流园区，争取开通更多至京、津、东北等地的始发高铁。确保北戴河机场二期6月30日前投入使用，促进北戴河机场对外通航能力有效拓展，空港物流园区建设步伐加快。推进京秦高速及北戴河新区支线等项目。完成北戴河机场快速通道建设和柳江地方铁路迁改工程，启动实施长途客运站搬迁、102国道绕城通道等工程，优化城市内循环。打通沿海公路冀辽界、秦唐界交通瓶颈。加快承秦铁路和秦承张通道建设前期工作。支持秦皇岛港转型升级，实施150航道改扩建，加快临港物流园区建设，完善港口集疏运配套体系，港口货物吞吐量完成1.9亿吨，集装箱55万标箱，谋划推进国际邮轮停靠港建设，加快打造国际化综合枢纽港。

（四）深入实施创新驱动战略，把牢增强核心竞争力的“金钥匙”。加快推进以科技创新为核心的全面创新，向创新要先进生产力、要核心竞争力，让创新真正成为推动秦皇岛跨越发展的不竭动力。

培育壮大创新主体。继续实施科技型中小企业成长计划，抓好苗圃、雏鹰、小巨人和上市四大工程，年内新增科技型中小企业400家、科技小巨人企业20家。加强知识产权保护，支持企业参与标准制定和发明创造，年内新增拥有自主知识产权的企业20家以上，培养专利优势企业10家以上，万人发明专利拥有量达到7件。

全面增强创新供给能力。以实施重大科技专项为抓手，围绕信息技术、先进装备制造、生命健康、新能源与节能环保、现代农业等重点领域，开展关键共性技术和核心技术攻关，提高产品质量和科技含量。加强高端装备产业技术研究院、农业科学研究院、环渤海生物产业研究院、海洋经济研究中心等建设，以学科链、专业链对接产业链，提升源头供给能力。

加快夯实创新载体。实施科技创新平台提升工程，落实企业研发费用税前加计扣除等政策，设立不少于1亿元的专项基金，引导企业加大技术创新投入，新增研发机构20家，25%的规模以上工业企业设立研发机构，力争更多国家级研发机构落户秦皇岛。推进秦皇岛高新区扩区工作，支持县区开展科技小镇、创新型县区建设。

充分激发创新创业活力。推进小微企业创业创新基地示范城市建设，大力推广众创、众包、众扶、众筹商业模式，支持建设各具特色的众创空间和孵化平台，打造面向大众的“双创”全程服务体系。积极与京津高层次人才薪酬、社保等制度接轨，对接省“百人计划”“百万燕赵工匠培养支持计划”，实施港城英才计划，吸引高层次创新团队和优质高校毕业生来秦创新创业，努力让创新的源泉充分涌流、创业的活力竞相迸发。

（五）切实加快改革开放步伐，增强内生发展动力。坚持上下联动、协同发力，投入更大精力抓好改革开放，更好发挥改革牵引作用和开放的带动作用，更多释放改革开放红利。

深化重要领域和关键环节改革。加快市以下政府间财政事权和支出责任划分改革，推进绩效预算管理改革。全面落实“营改增”任务，推进结构性减税、普遍性降费和水资源费改税等试点工作。支持地方金融业健康快速发展，推进秦皇岛银行增资扩股，力争年底前秦皇岛市农商银行实现挂牌开业；继续推进“风险补偿资金”和“应急转贷资金”制度，推动“政银保”合作。深化国有企业改革，开展国有资本优化重组，强化对党政机关、事业单位所属企业国有资本的监管。继续推进水、电、气等资源性产品价格改革。大力推动商事制度、事业单位、机关内设机构、海洋管理体制、科教文卫和安全生产领域等各项改革举措落地，下决心打破“中梗阻”，打通“最后一公里”。

积极主动扩大对外开放。发挥好投资促进、企业服务、政务服务“三个中心”作用，落实招商引资目标任务分解和考核奖惩办法，提升招商引资精准度和实效性，努力拓展开放空间和深度。不断延伸秦皇岛港服务链条，拓展港口腹地，健全口岸查验配套设施，推进电子口岸平台体系建设，优化物流、加工、结算、出入境等服务。争取设立综合保税区。推进煤炭交易市场建设，加快成品油、煤炭、粮食等大宗商品交易中心建设，充分发挥进境水果口岸的带动作用，努力建设北方地区进口水果集散地。

推动民营经济转型发展实现突破。认真落实鼓励民营经济发展的政策措施，强化公共服务，搭建企业间互动交流平台，帮助企业解决好生产经营中的实际困难。坚决放开市场准入，对法律法规没有明确禁止的行业和领域，鼓励和支持民间资本进入；对可以采用市场化运作的基础性公共项目，向民间资本全面开放。引导民营企业调整资金投向、发展新兴产业、加快转型升级。关心爱护企业家，保护企业家精神，搞好企业家培训，支持企业家专心创新创业，促进民营经济“万木丛生”、茁壮成长，力争市场主体突破19万户，民营经济增加值增长7%以上。

营造良好营商环境。开展优化营商环境专项行动，以“零容忍”的态度，整治“潜规则”、效率低下等“顽疾”，凡是百姓反映集中的突出问题，必须第一时间介入；凡是企业反映强烈的突出问题，必须严肃查处；凡是社会反映普遍的突出问题，必须彻底解决。加强政务诚信建设，严格履行各项约定义务，打造“诚信秦皇岛”品牌。动员全社会参与，加强社会监督，敢于揭短亮丑，倾力打造开放包容、互利合作、亲商安商的良好环境，真正使秦皇岛成为投资置业的热土。

（六）加大生态环境保护治理力度，建设天蓝地绿水秀的美丽港城。把生态环保作为发展坚守底线，科学施策、标本兼治、铁腕出击，让人民群众享有更多的蓝天白云、绿水青山。

开展“蓝天行动”。突出精准治霾，设立13.6亿元蓝天基金，落实好省大气污染防治“1+18”工作方案，坚决控制污染物排放。深化工业企业治理，淘汰城市建成区内35蒸吨及以下，城郊、县城、乡镇10蒸吨及以下燃煤锅炉，强力推进清洁能源替代散煤，彻底解决散煤污染问题。开展保留燃煤锅炉治理设施提标改造，实施重点行业工业企业深度治理，深化挥发性有机物治理，彻底关停不符合产业政策的“小散乱污”企业，坚决打好打赢“蓝天保卫战”。

实施“碧水行动”。完善河长制责任体系，系统推进水污染防治和水生态保护，实施城市区污水调配和护城河水体整治工程，完成大马坊河下游段水生态修复，集中治理食品加工、甘薯加工等行业废水污染，全部搬迁或关闭禁养区内的养殖企业，确保城市建成区河流消除黑臭水体，入海河流水质持续好转，近岸海域水质全面达标。推进城镇污水处理厂扩容、提标改造和工业聚集区污水集中处理设施建设。完成县城以上集中式饮用水源地保护区划定，实施水源地一级保护区封闭隔离工程，确保城市集中饮用水水源地水质达标率100%。实施“蓝色海湾行动”，推行“滩长制”，让每一段沙滩都有人管、管得好。大力整治海域秩序环境，严厉打击非法开采海砂等行为，积极创建国家海洋公园。

推进“净土行动”。制定土壤污染防治工作实施方案，推进工业大宗固废和尾矿库资源化利用。鼓励土壤污染第三方治理，探索建立政府出政策、社会出资金、企业出技术的土壤污染治理与修复市场化机制。强化工矿企业环境监管，严厉打击随意倾倒、堆放危险废物行为。通过测土配方施肥等措施，控制农业面源污染，减少化肥农药施用量。落实企业治理土壤污染的主体责任，扎实推进露天矿山开采退出和矿山生态修复，年内关停尾矿库39座。

打造“森林城市”。落实国家储备林项目，实施“一心两翼十带多园”建设工程，推进国家园林县城创建工作，确保完成造林绿化50万亩，城市绿化覆盖率、绿地率分别达到41.25%和39.69%，争创国家森林城市。完善沿海防护林和湿地长效保护机制。加强森林火灾预防和扑救工作。完成第三届省园博会主体工程，建设一个气势恢弘、精品荟萃、特色鲜明的现代化园博园。

（七）统筹城乡区域发展，加快新型城镇化步伐。对标世界一流滨海城市，以全国文明城市、国家卫生城市、国家森林城市“三城同创”为动力，建设新型城镇化与城乡统筹示范区。

提高规划建设管理水平。完成新一轮城市总体规划编制，加快推进“多规合一”，实现“一张图”管理。实施生态修复城市修补工程，启动石河整治二期、石河与引青济秦供水管线连接工程。以秦皇岛开发区、北戴河新区为重点，科学推进地下管廊、海绵城市建设。改造提升范家店片区，加快开发艺术中心及周边片区、海阳片区、道南片区，完成山海关火车站子站房和南广场建设。推进智慧城市建设，实现主城区及县城重点区域免费WIFI全覆盖。引进卓越绩效管理模式，理顺市区两级城市管理综合执法责任，大力开展“全民洗城”、交通拥堵治理、“无双违县区”创建等活动，城市生活垃圾无害化处理率100%，打造全国最干净城市。

推动县城扩容提质。深入推进县城建设三年攻坚行动，加快完善水、电、气、热、讯等基础设施，推进公共服务向县城延伸，年内每个县完成2条城市景观路综合整治，县城路网密度达到7公里/平方公里以上，洁净供热率达到75%。启动抚宁康养职教城项目，推进教育资源整合集聚，促进卢龙、昌黎、青龙全部高中进县城，加快产城教融合发展。

抓好特色小镇和美丽乡村建设。落实省特色小镇建设指导意见，支持石门镇建设国家特色小城镇，推动靖安镇、祖山镇、榆关镇等小城镇加快发展，培育北戴河新区医疗康养旅游小镇、昌黎县葡萄酒小镇等一批新兴特色产业小镇。坚持以镇带业、以业兴镇，加强县域园区承载能力建设，推动小城镇产城融合发展，加快县域经济增比进位。抓好1个省级片区、227个省级重点美丽乡村建设，加强历史文化名镇名村、传统村落、历史建筑的保护开发，打造既有现代文明、又具田园风光的“高颜值”特色乡村。

引导人口有序转移。支持卢龙县创建国家级新型城镇化综合试点地区，探索构建农业转移人口市民化成本分担机制和多元化投融资机制。实施农民工职业技能培训提升计划，完善农村土地承包经营权、宅基地使用权、集体收益分配权退出机制，保障进城落户人员公共服务落实到位，提高农村人口向城镇转移的主动性。推进乡改镇、镇改街、村改居。深化户籍制度改革，制定更加积极的政策，广聚贤才来秦施展才华。

（八）着力保障改善民生，加快补齐社会事业短板。为政之要，民生至上。我们一定始终把群众冷暖放在心头，时刻把改善民生责任扛在肩上，切实把惠民利民举措落到实处，让发展更有“温度”、幸福更有“质感”。

竭诚办好民生实事。立足于让人民群众更多共享发展成果，坚持尽力而为、量力而行原则，从解决与群众生产生活密切相关的突出实际问题入手，精心谋划实施推进交通便民、扶助困难群体、提升公共服务等6个方面20件民生实事，集中财力人力，逐级落实责任，明确目标要求，强化跟踪督导，切实把该办的事竭力办好，做出的承诺说到做到，努力让民生实事真正深入人心、温暖民心。

坚决打好脱贫攻坚战。今年是我市脱贫攻坚的决战决胜之年，要继续实施基础设施改善、产业就业脱贫等八大工程，夯实脱贫攻坚基础；探索有效途径，不断壮大村级集体经济实力；统筹整合涉农资金，实施“政银企户保”金融扶贫；加强与中央和省帮扶单位对接沟通，深入开展“百企帮百村”精准扶贫行动，争取多层次力量支持扶贫；充分发挥基层党组织、帮扶单位、驻村工作组作用，增强贫困群众内生动力，压实责任、找准路子，扶到点上、扶到根上，确保青龙成功摘帽，确保小康路上不丢一户、不落一人。

认真做好就业创业工作。重点抓好高校毕业生、农村转移劳动力、退伍军人等人群就业。完善市职业技能公共实训基地配套，推动高校毕业生就业促进计划和创业引领计划有效落实。健全创业扶持政策体系，加强创业园区建设，持续推进创业带动就业。规范津贴补贴管理，推进绩效工资改革，提高机关事业单位工资收入水平。及时发布企业工资指导线和指导价位，依法治理拖欠农民工工资问题。发挥好失业保险援企稳岗作用。年内城镇新增就业4.9万人，农村劳动力转移就业1.7万人，努力让人民在劳动中创造财富，在奋斗中实现人生价值。

织牢社会保障安全网。深入推进社保扩面提标工程，全面实施城乡居民基本医疗保险制度，城乡居民基础养老金标准提高到90元/月。加快推进老旧小区改造提升、棚户区改造和农村危房改造，完成“两租房”分配入住2000套。完善综合救助体系，加强低保线与扶贫线“两线合一”动态管理，继续提高城市和农村最低生活保障标准，坚决把基本民生的底线兜住兜牢，让困难群众心里有温暖、生活有奔头。

全面发展社会事业。坚持教育优先发展地位，新建幼儿园16所、小学8所、初中6所，妥善解决青龙偏远地区教学点问题；加快普通高中布局调整，推进职教园区项目建设，支持燕山大学等高校争创“双一流”大学；实施师德建设工程，办好人民满意的教育。推进医疗服务水平提升和布局优化调整，加快广济医院、北戴河国际肿瘤医院、北医三院北戴河国际医院等建设，推动市第一医院综合门诊楼如期开工，市精神卫生中心竣工使用；深化县级公立医院综合改革，推进城市公立医院综合改革，完善分级诊疗和双向转诊就医格局。加快构建现代公共文化服务体系，推进市博物馆、市规划馆等重点公共设施建设。广泛开展群众性文化活动，打造“春夏秋冬”四季特色文化品牌。加强诚信体系建设，提高市民文明素质和城市文明程度，确保以优异成绩进入全国文明城市行列。广泛开展全民健身，促进健康秦皇岛建设，使更多人享受运动快乐、拥有健康体魄。

确保社会和谐稳定。大力开展食品药品安全专项整治，确保人民群众饮食用药安全，创建国家食品安全城市。强化安全生产风险防控和隐患排查治理，深化高危行业和领域专项整治，保持“打非治违”高压态势，确保全年不发生重大及以上安全生产事故。加强灾害救助应急反应和保障能力建设。全面落实社会稳定风险评估措施，做好矛盾纠纷排查调处工作，依法严厉打击各类违法犯罪活动，强化治安和特殊商品管控，全方位提高人民群众安全感，争创全国综治优秀城市“六连优”。

深入实施“两河战略”，高标准做好旅游旺季服务保障工作。加强国防教育和国防后备力量建设，支持部队深化改革，深入开展双拥共建活动，推进军民融合发展。支持工会、共青团、妇联、科协等人民团体发挥桥梁纽带作用，做好新闻出版、广播电视、老龄、残疾人、妇女儿童、气象、防震减灾、民族宗教、外事侨务、人民防空、邮政通信、审计、统计、地方志、档案和援疆等工作，推动各项事业取得新进步。

四、全面加强政府自身建设

打铁还需自身硬。落实各项目标任务，需要我们以更高标准、更严要求、更实作风，扎实推进法治政府、廉洁政府、服务政府建设，不断提高政府治理能力和现代化水平。

（一）坚持以严为基，压实全面从严治党责任。深入贯彻党的十八届六中全会精神，推动“两学一做”学习教育常态化、制度化，牢固树立“四个意识”，坚决维护以习近平同志为核心的党中央权威，自觉在思想上政治上行动上同党中央保持高度一致。严格执行《关于新形势下党内政治生活的若干准则》和《中国共产党党内监督条例》，落实“一岗双责”，强化主体责任，加大行政监察和审计监督力度，强化廉政风险防控，保持惩治腐败高压态势，用党风廉政建设和反腐败工作新成效，促进经济社会新发展。

（二）坚持以民为本，加快推进政府职能转变。坚持问政于民、问计于民、问需于民，畅通群众意愿表达渠道，虚心听取各界意见建议。深化“放管服”改革，完善市级行政权力清单、责任清单和负面清单制度，全面推行“双随机、一公开”监管。深化行政审批制度改革，组建市行政审批局，推进“互联网+政务服务”，推动网上政务大厅和实体政务大厅融合发展。开展减证便民专项行动，全面清理并取消各种不合理证明和手续，让政务服务更加智能便捷、公平可及。

（三）坚持以法为纲，切实提高执行力和公信力。自觉运用法治思维和法治方式开展工作，推动法律顾问制度全覆盖。健全依法科学民主决策机制，努力控制决策风险。加强政府行政立法，提高立法质量。严格规范公正文明执法，全面落实行政执法公示、执法全过程记录、重大执法决定法制审核制度。深入实施“七五”普法规划，营造尊法学法守法用法的浓厚氛围。自觉接受人大及其常委会的法律监督和工作监督、政协的民主监督，接受社会和舆论监督，深入推进政务公开和政府信息公开，确保所有工作都能体现人民意愿、维护人民利益、接受人民监督。

（四）坚持艰苦奋斗，驰而不息改进工作作风。严格落实中央“八项规定”精神，持续开展“四风”整治。强化机关作风整顿，深入落实“五到四从四多”工作法、“两式两化”和工作日志等制度，倡导夙兴夜寐、激情工作的风尚。建立健全正向激励机制和容错纠错机制，不断完善责任机制和督查机制，切实加强绩效管理考核，让优秀成为习惯，担当成为时尚。发扬艰苦奋斗精神，大力精简经费开支，一般性财政支出压减5%以上，真正把有限的资金用在改善民生和发展经济上，用政府的“紧日子”，换来百姓的“好日子”。

各位代表！使命重在担当，实干成就梦想。让我们更加紧密地团结在以习近平同志为核心的党中央周围，在省委省政府和市委的坚强领导下，凝神聚力，同心同德，以更加饱满的热情、更加昂扬的斗志、更加务实的作风，撸起袖子加油干，为夺取全面建成小康社会伟大胜利，为加快建设沿海强市、美丽港城和国际化城市而不懈奋斗！
